# Evaluating the evidence for models of life course socioeconomic factors and cardiovascular outcomes: a systematic review

**DOI:** 10.1186/1471-2458-5-7

**Published:** 2005-01-20

**Authors:** Ricardo A Pollitt, Kathryn M Rose, Jay S Kaufman

**Affiliations:** 1 Department of Epidemiology, School of Public Health, University of North Carolina at Chapel Hill, Chapel Hill, North Carolina, USA

## Abstract

**Background:**

A relatively consistent body of research supports an inverse graded relationship between socioeconomic status (SES) and cardiovascular disease (CVD). More recently, researchers have proposed various life course SES hypotheses, which posit that the combination, accumulation, and/or interactions of different environments and experiences throughout life can affect adult risk of CVD. Different life course designs have been utilized to examine the impact of SES throughout the life course. This systematic review describes the four most common life course hypotheses, categorizes the studies that have examined the associations between life course SES and CVD according to their life course design, discusses the strengths and weaknesses of the different designs, and summarizes the studies' findings.

**Methods:**

This research reviewed 49 observational studies in the biomedical literature that included socioeconomic measures at a time other than adulthood as independent variables, and assessed subclinical CHD, incident CVD morbidity and/or mortality, and/or the prevalence of traditional CVD risk factors as their outcomes. Studies were categorized into four groups based upon life course design and analytic approach. The study authors' conclusions and statistical tests were considered in summarizing study results.

**Results:**

Study results suggest that low SES throughout the life course modestly impacts CVD risk factors and CVD risk. Specifically, studies reviewed provided moderate support for the role of low early-life SES and elevated levels of CVD risk factors and CVD morbidity and mortality, little support for a unique influence of social mobility on CVD, and consistent support for the detrimental impact of the accumulation of negative SES experiences/conditions across the life course on CVD risk.

**Conclusions:**

While the basic life course SES study designs have various methodologic and conceptual limitations, they provide an important approach from which to examine the influence of social factors on CVD development. Some limitations may be addressed through the analysis of study cohorts followed from childhood, the evaluation of CVD risk factors in early and middle adulthood, and the use of multiple SES measures and multiple life course analysis approaches in each life course study.

## Background

The adult behavioral lifestyle theory of cardiovascular disease (CVD) describes an adult's lifestyle choices and levels of physiologic risk factors as the primary predictors of CVD risk [1-3]. This approach is supported by a relatively consistent literature demonstrating an inverse, graded relationship between SES and CVD [4-12]. There is also a growing literature that focuses upon the influence of SES at different points in life on adult CVD risk.

A force behind the interest in the impact of early-life conditions on adult health is the fetal origins (Barker) hypothesis. This hypothesis, which has been met with considerable support and criticism, posits that poor nutrition during fetal and early infant development ("critical periods") can increase risks for adult disease. While the dominance of the adult lifestyle model of chronic disease development may not be threatened by evidence for the fetal origins hypothesis, a reconsideration of the primary importance of adult behaviors and risk factors is underway.

A life course approach to chronic disease proposes that the combination, accumulation, and/or interaction of the social environments and biological insults experienced throughout the life course impact current and future events, environments, and health conditions and thus ultimately impact adult health [13,14]. Various interrelated theories have been put forward, [14-23] and many study designs have been utilized to examine the impact of life course SES [13,17,19,23-30].

This review describes the major groups of conceptual life course SES models, and then categorizes and summarizes studies that examine the associations between life course SES and CVD risk. Studies are grouped by the basic life course design utilized; summaries include methodologic critiques and descriptions of certain key studies. Evidence supporting each conceptual life course model is considered and future research directions are discussed.

### Life course SES conceptual models

The different extant hypotheses on the influence of life course SES on CVD can be grouped into four broad conceptual models: the latent effects, pathway, social mobility, and cumulative life course models. Most studies reviewed tested the influence of life course SES on CVD outcomes via the operation of one or more of these models.

#### The latent effects model

The "latent effects" life course conceptual model hypothesizes that adverse early life experiences increase the risk of CVD in later life, independent of intervening SES, lifestyle, or traditional CVD risk factors. Power & Hertzman (1997) describe a latent effects model wherein certain early life events may have strong independent effects on adult health [14]. Kuh and Ben-Schlomo (1997) propose the related concept of biological chains of risk, wherein prenatal and early life socioeconomic factors affect biologic resources and directly influence adult health [13]. A recent formulation suggests the operation of biological or developmental influences during early "sensitive periods" which permanently impact the organism [28].

#### The pathway model

In this life course model, early life events and environments influence later life experiences, opportunities, and health risk factors. Hertzman et al. (2001) propose a developmental process linking early-life psychosocial environments with adult health risk via pathway effects, wherein early experiences place an individual onto a certain "life trajectory," eventually impacting adult health [28]. Similarly, Kuh et al. (1997) use the concept of "social chains of risk," whereby early events influence later life experiences, thus impacting adult disease risk [20]. Blane (1999) describes an "ongoing social process" wherein a continuity of social circumstances are linked and may create a "chain of disadvantage [31]."

While a pathway life course model is intuitively appealing, its operation is difficult to test empirically. Life course studies typically collect information on participants at two or three time points, which does not permit the continuous, lifelong operation of pathway effects to be observed.

#### The social mobility model

Social mobility theories all hypothesize that SES mobility across the life course impacts adult health, although the different proposed theories posit different health effects. Forsdahl (1978) hypothesized that deprivation in early life followed by later affluence combine to produce elevated CHD mortality risk, partly via elevation of adult cholesterol levels [32]. Others proposed that natural "health selection" occurs, wherein less healthy individuals tend to have downward social mobility and healthier individuals tend to be upwardly mobile [30,33]. In contrast, the "health constraint" hypothesis contends that socially mobile individuals possess health characteristics of both the SES group they leave and the one they join, so that social mobility minimizes the health differences between SES groups [15,34].

#### The cumulative model

A cumulative SES life course model hypothesizes that psychosocial and physiological experiences and environments during early and later life accumulate to influence adult disease risk. Davey-Smith et al. (2002) suggest that if factors operating at different life stages are combined, large differences in CVD risk will be observed [35]. Kuh et al. (1997) describe an individual's biological resources accumulated over the life course as their 'health capital' [20], which describes and influences current and future health. The "accumulation of risk" model described by Ben-Schlomo and Kuh (2002) proposes that the impacts of different life course events accumulate but do not interact [26].

## Methods

We conducted a MEDLINE search using keywords and MeSH terms related to SES, life course, early life, longitudinal studies, CVD and CVD risk factors. References identified and books on life course research were also examined for study citations. Inclusion criteria were: (1) publication date between January 1966 and July 2003; (2) SES or related measures as independent variables; (3) outcomes of subclinical CHD, CVD morbidity and/or mortality, or traditional CVD risk factors. Behavioral risk factors were limited to: adult diet, physical activity, alcohol consumption, and smoking. Physiological outcomes were: measures of obesity, blood pressure, intima-media thickness, fibrinogen, insulin resistance, dyslipidemia, and lung function. Ecologic studies, studies primarily evaluating the fetal origins hypothesis, and studies only examining the impact of birth weight, height, upper leg length, patterns of growth, household crowding, geographic mobility or numbers of siblings were excluded. Forty-nine studies met the inclusion criteria.

Quantitative summarization of the study findings is problematic due to varied study populations, SES measures and CVD outcomes evaluated, time points evaluated, and designs utilized. Moreover, a tally approach to summarizing across studies based on statistical significance can be misleading due to the different strengths, weaknesses, power and biases of each study (p. 671) [36]. Summaries therefore consist primarily of qualitative information on the types and directions of associations observed and the findings reported by authors. Although we avoid focusing on statistical significance, in most studies the authors' decision to declare a positive finding was tied to a null-hypothesis significance test using a p-value of < 0.05.

### Categorization of life course studies by life course study design

Studies were categorized by their life course study design(s) and study question(s) into four groups: early SES → outcome, early SES → risk factor, social trajectory, and cumulative SES studies. Several studies appear in multiple categories, as they employed more than one of these designs. Table 1 describes the research questions and hypotheses typically considered by each design.

**Table 1:** Life course study designs: hypotheses tested and typical study questions posed

**Life course study design**	**Typical chronological analysis set-up used in study design**	**Typical study question**
Early SES → Outcome	Early-life SES variable(s) used to predict CVD (e.g., CHD, stroke). Adjusted for later-life events, behaviors, risk factor levels to determine "direct" effect of early life SES.	Is there a significant independent effect of early-life (childhood) SES on the adult risk of CVD after adjusting for later-life SES and risk factors?
Early SES → Risk Factor	Early-life SES variable(s) used to predict adult CVD risk factors levels.	How does early-life SES affect later-life levels of behavioral and physiological CVD risk factors?
Social Trajectory	Inter- or intragenerational movement from one SES level to another (i.e., Low SES to High SES) used to predict adult CVD risk factors levels or CVD outcomes.	How does social mobility from one point to another during the life course affect the risk of CVD?
Cumulative SES	A summary variable indicating number of negative SES events/environments over the life course used to predict adult CVD risk factors levels or CVD outcomes.	How does the accumulated number of negative SES-related exposures across the life course influence the risk of CVD?

CHD = Coronary heart disease.

## Results

### Early SES → outcome life course studies: overview

These studies examined the effect of childhood and/or adolescent SES on risk of adult CVD (e.g., incident myocardial infarction (MI), CVD death, incident/fatal stroke) [37-56] and typically tested the latent effects hypothesis (i.e., examining the "direct" effect of early-life SES on adult CVD risk). They commonly measured the independent effect of childhood SES by statistically adjusting for later-life SES and CVD risk factors in regression models.

### Early SES → outcome studies: summary of results

Table 2 lists these 20 studies according to CVD outcome utilized (see Additional file 1 for greater detail on these studies). All 19 studies conducting unadjusted or age-adjusted analyses reported a point estimate consistent with an inverse association between early-life SES and risk of one or more of the adult cardiovascular outcomes. Authors of 14 studies concluded that their findings supported some or all of their study hypotheses [39,41-45], 47[49-55]. Fourteen of 16 studies adjusting for adult SES and/or CVD risk factors reported some indication of an inverse association; however, in only five did the authors suggest that their results support the hypothesis of an inverse association between early life SES and CVD.

**Table 2:** Summary of SES – CVD life course studies using an early SES → outcome design

**1^st ^Author, year & reference number** **Study name**	**Early life SES measures**	**Variables adjusted for other than age**
**Acute/ Survived MI, CVD**		
Burr 1980 [49] South Wales hospital cohort	Father's occup (RG, 3 groups), father unemployed (> 1 year), family size	Current SES
Notkola 1985 [40] East-West Study	5-level index: father's occup & farm size	Current SES, CVD RF's
Coggon 1990 [37] Stoke-on-Trent & Newcastle study	Father's occup (RG, 5 groups), height, sibling death	Current SES, smoking
Hasle 1990 [48] Danish worker's union study	8 variables (yes/no) on parent's occup, health, household, residence, edu, illness	None
Kaplan 1990 [43] Kuopio Study	Factor analysis of edu, occup, farm, farm size, perceived wealth	CVD RF's
Lundberg 1993 [51] Swedish population cohort study	4 yes/no variables: economic hardship, large family, broken family, dissension	Current SES, gender
Gliksman 1995 [39] Nurses' Health Study	Father's occup at 16 years	Current SES, CVD RF's
Lamont 2000 [41] Newcastle 1,000 Families Cohort	Birth: father's occup; 5 & 10 years: parent's occup, housing, # of adverse life events	Current SES, CVD RF's
Marmot 2001 [46] Whitehall II Study	Childhood: father's occup (RG, 4 groups), age at leaving edu Labor force entry: Occup	Current & child SES
Wamala 2001 [45] Stockholm Study	Early-life SES disadvantage index (0–3) of 3 variables: large family, born last, low edu	Current SES, CVD RF's
**Stroke**		
Gliksman 1995 [39] Nurses' Health Study	Father's occup (4 groups) at 16 years	Current SES, CVD RF's
Coggon 1990 [37] Stoke-on-Trent & Newcastle study	Father's occup (RG, 5 groups), height, sibling death	Current SES, smoking
Davey Smith 1998 [44] Collaborative Study	Father's occup (RG, 4 groups), also mnl vs. non-mnl	Current SES, CVD RF's
Frankel 1999 [54] Boyd Orr Cohort	Father's occup (RG, 5 groups)	Townsend area deprivation score
Dedman 2001 [56] Boyd Orr Cohort	Persons/room, tap water (yes/no), toilet type, ventilation, cleanliness (3 levels each)	Childhood SES, area deprivation score
**CHD Mortality**		
Notkola 1985 [40] East-West Study	5-level index using father's occup & farm size	Current SES, CVD RF's
Lynch 1994 [38] Kuopio Study	SES index (3 groups), by parents' edu, occup, farm, perceived wealth	Current SES
Vagero 1994 [50] Uppsala Birth Cohort Study	Occup of head of household (mnl, non-mnl, unemployed)	Current SES
Gliksman 1995 [39] Nurses' Health Study	Father's occup (4 groups) at 16 years	Current SES, CVD RF's
Davey Smith 1998 [44] Collaborative Study	Father's occup (RG, 4 groups), also divided into mnl vs. non-mnl	Current SES, CVD RF's, area deprivation
Hart 1998 [42] Collaborative Study	Early SES: father's occup; Labor force entry: Occup; At screening: occup	None
Frankel 1999 [54] Boyd Orr Cohort	Father's occup (RG, 5 groups)	Townsend area deprivation score
Davey Smith 2001 [47] Glasgow Alumni Cohort	Father's social class, (RG, 5 groups)	CVD RF's
Dedman 2001 [56] Boyd Orr Cohort	Persons/room, tap water (yes/no), toilet type, ventilation, cleanliness (3 levels each)	Childhood SES, Townsend area deprivation score
Davey Smith 2002 [52] Collaborative Study	Father's occup (mnl/non-mnl)	Current SES, CVD RF's
Claussen 2003 [53] Oslo Mortality Study	Index of housing conditions items	Current SES
Osler 2003 [55] Project Metropolit	Father's social class (3 groups) by occup	Birth weight, IQ at age 12

CHD = Coronary heart disease; Edu = Education; IHD = Ischemic heart disease; MI = Myocardial infarction; Mnl = Manual occupational class; Non-mnl = Non-manual occupational class; Occup = Occupation; RF = Risk factor; RG = Registrar General's social class categories.

For studies with CHD and stroke outcomes limited to early-life SES (i.e., not controlling for adult SES or other risk factors) almost all included a point estimate consistent with an inverse association. However, in adjusted analyses, authors of only one study with a CHD-related outcome and fewer than half the studies of CVD mortality reported inverse adjusted associations. Thus, the existence of a "direct effect" of early-life SES after adjusting for adult risk factors was not strongly supported.

### Early SES → outcome studies: methodologic issues

To prevent statistical confounding by adult conditions, most studies employed statistical models that adjusted the association between childhood SES and adult CVD risk for adult SES and behavioral/ physiological CVD risk factors. This may be an over-adjustment, as certain risk factors (e.g., BMI, smoking) are part of the pathways through which low childhood SES may influence CVD risk. Little information is usually available on how participants' early-life SES may have influenced levels of adult CVD risk factors. Additionally, if unknown variables influence both the distribution of a CVD risk factor adjusted for and the distribution of the outcome measure, then adjustment will result in an incorrect partitioning of the total effect of early-life SES into "direct" and "indirect" components [57-59]. Thus, early SES → outcome studies which seek to determine the "direct," adjusted effect of early-life SES on CVD risk may incorrectly estimate this effect [60], leading to questions about the accuracy of such estimates.

Some studies examined and qualitatively compared the effects of adjustment for different classes of CVD risk factors. Gliksman et al. (1995), for example, analyzed the association between father's occupational class and adult CVD in a series of models adjusting for different classes of potential mediators or confounders. This allowed for a more complete description of the impact of adult environment, behavior, and physiologic risk factors versus the latent impact of early life socioeconomic factors [39]. Studies utilizing more than one life course study design ("mixed-design studies") take a related approach and are considered in the Discussion section.

### Early SES → risk factor studies: overview

Life course studies in the early SES → risk factor group evaluated the influence of early- and/or mid-life SES or living conditions on later-life behavioral or physiologic CVD risk factors [14,40,44,52,61-73]. These designs are often similar to those used in the early SES → outcome studies group except that CVD risk factors are the outcomes. The life course hypotheses typically examined, however, include both the latent effects and pathway conceptual models. The typical study design involves a statistical model where early-life SES measures predict the levels of CVD risk factors in adulthood.

### Early SES → risk factor studies: summary of results

Table 3 outlines the 17 studies reviewed (see Additional file 2 for detailed summaries). Five of six studies reported associations between low early-life SES and little or no adult leisure-time physical activity [14,61,63,64,66], five of five found associations with high adult alcohol intake [52,61,63,68,73], and eight of twelve studies reported associations with higher smoking rates as study findings [14,44,52,63,65-68]. Nine of 11 studies reported (unadjusted) associations between lower early-life SES and elevated BMI or WHR [14,44,63,64,67,68,70,71,73]. The impact of a statistical adjustment for other CVD risk factors varied, including no impact [59], attenuation with a persistence of the significant effect [46,55], and strong attenuation with associations no longer apparent [54,78].

**Table 3:** Summary of SES – CVD life course studies using an early SES → risk factor design

**1^st ^Author, year & reference number ** **Study name**	**Early life and/or adult SES measures**	**Variables adjusted for other than age**	**CVD risk factor(s) measured**
Arnesen 1985 [65] Tromso Heart Study	Early-life: 4-level index of household economic conditions	CVD RF's	Cholesterol, SBP, glucose, BMI, smoking, more
Notkola 1985 [40] East-West Study	Early-life: 5-level index using father's occup & farm size; Adult: occup (6 groups)	None	Smoking, cholesterol, SBP
Wadsworth 1985 [98] Medical Research Council National Survey of Health Study	Early-life: index of father's occup & parents' edu; Adult: occup (RG) & employment	CVD RF's	SBP, DBP
Braddon 1986 [71] British 1946 Birth Cohort	Early-life: 2 social class indices of father's occup; Adult: occup (RG, 8 groups), edu (high/low)	Adult SES, CVD RF's	Obesity (BMI > 30.0 for men, > 29.1 for women)
Peck 1994 [66] Swedish census cohort study	Early-life: father's occup (7 groups); Adult: occup (7 groups)	None	Smoking, physical activity
Blane 1996 [64] Collaborative Study	Early-life: father's occup (RG, 4 groups); Adult: occup (RG, 4 groups)	Adult SES	DBP, physical activity, smoking, BMI, FEV1, more
Lynch 1997 [99] Kuopio Study	Child: SES index (3 groups); Adolescent: edu (3 groups); Adult: occup, income, possessions, more	Energy intake	Smoking, drinking, obesity, physical activity, diet
Power 1997a [14] 1958 British Birth Cohort	Child: father's occup (RG, 4 groups); At 23 years: occup, edu; At 33 years: occup	None	BMI (obesity)
Power 1997b [90] 1958 British Birth Cohort	Child: father's occup (RG, 4 groups); At 23 years: occup, edu; At 33 years: occup	None	Smoking, BMI (obesity)
Davey Smith 1998 [44] Collaborative Study	Early-life: father's occup (4 groups); Adult: occup (6 groups)	None	Smoking, DBP, cholesterol, BMI, FEV1
van de Mheen 1998 [63] Longitudinal Study, Netherlands	Early-life: father's occup (6 groups); Adult: occup (6 groups)	Adult SES	BMI, smoking, alcohol, leisure physical activity
Brunner 1999 [67] Whitehall II Study	Early-life: father's occup (RG, 4 groups); Adult: occup (Civil Service grade, 4 groups)	Adult SES	Smoking, activity, HDL, BMI, fibrinogen, more
Davey Smith 2002 [52] Collaborative Study	Early-life: father's occup (mnl/non-mnl); Adult: occup (mnl/non-mnl)	None	Smoking, alcohol, area deprivation
Lawlor 2002 [68] British Women's Heart Study	Early-life: father's occup (RG, 6 groups); Adult: current occup (RG, 6 groups)	Adult SES	Insulin resistance, SBP, cholesterol, BMI, smoking, triglycerides, alcohol, more
Poulton 2002 [73] Dunedin Multidisciplinary Study	Early-life: parental occup (6 groups) at 0, 3, 5, 7, 9, 11, 13 & 15 years; Adult: current occup (3 groups)	Adult SES	BMI, WHR, SBP, smoking, alcohol, more
Lawlor 2003 [69] British Women's Heart Study	Early-life: father's longest occup (mnl / non-mnl); Adult: longest occup (RG, 6 groups)	CVD RF's	HOMA, SBP, HDL, triglycerides
Parker 2003 [70] Newcastle 1000 Families Study	Birth: father's occup & housing; 5 & 10 years: same, plus adverse life events; Adult: wage earner's occup	None	CMS, BMI, WHR, fasting insulin, triglycerides, HDL

BMI = Body mass index; CMS = Central metabolic syndrome; DBP = Diastolic blood pressure; Edu = Education; FEV1 = Forced expiratory volume in 1 second; HOMA = Homeostasis model assessment score; Mnl = Manual occupational class; Non-mnl = Non-manual occupational class; Occup = Occupation; RF = Risk factor; RG = Registrar General's social class categories; SBP = Systolic blood pressure; WHR = Waist-to-hip ratio.

### Early SES → risk factor studies: methodologic issues

As with the early SES → outcome studies, concerns that lack of adequate adjustment or over-adjustment may lead to biased estimates are germane to this group of studies. The issue of covariate adjustment is complex in these studies, given that many CVD risk factors are considered. Certain physiologic risk factors (e.g., insulin resistance, dyslipidemia) may be linked to early-life SES through latent physiologic effects of negative childhood exposures, continuous exposure to negative physiologic stimuli, or other physiologic or life course pathway mechanisms [14,63,70,74]. In contrast, the links between early-life SES and levels of behavioral risk factors (e.g., smoking, diet), and certain physiologic risk factors (e.g., BMI, hypertension), may operate principally through learned behaviors, behavioral responses to negative psychosocial stimuli, or other psychosocial life course pathway effects. The associations may develop through the operation of multiple physiologic and psychosocial pathways, making "correct" covariate adjustment challenging within a single model. Thus, statistical adjustment should take into account the specific life course pathways hypothesized to be operating [39]. Previous findings should be used to generate a priori hypotheses; nonetheless, there is considerable opportunity for inappropriate adjustments to be made.

### Social trajectory studies: overview

Studies evaluating the social mobility life course model typically considered the impact of inter-generational or intra-generational social mobility on CVD and CVD risk factors [38,44,49,64,72,73,75-79]. Inter-generational mobility was usually determined by contrasting the participant's father's occupational SES to the participants'. Intra-generational SES was typically defined as a change in occupational SES from early adulthood to later adulthood. Six of 11 studies evaluated adult CVD risk factors as outcomes [49,64,72,73,75,77]; seven evaluated CVD mortality or CHD [38,44,75-79]. Four adjusted only for age [38,49,76,80].

### Social trajectory studies: summary of results

Table 4 outlines these studies (see Additional file 3 for greater detail). Of 10 studies carrying out statistical analyses [38,44,49,72,73,75-79], six did not report associations between upward or downward mobility and either elevated levels of CVD risk factors or increased CVD morbidity or mortality when compared to stable low-SES or high-SES trajectories [38,44,49,75,78,79]. Nine studies, however, reported the suggestion of inverse, although not always statistically significant, relationships between social mobility and a CVD-related outcome [38,44,72,73,75-79]. Two studies found marked differences in CVD mortality risk between upwardly mobile individuals and individuals maintaining the same SES across time. However, one study reported increased CVD risk among the upwardly mobile [77] and one reported decreased mortality risk [79].

**Table 4:** Summary of SES – CVD life course studies using a social trajectory design

**1^st ^Author, year & reference number ** **Study name**	**SES measures**	**Variables adjusted for other than age**	**CVD risk factor(s) / outcomes measured**
Kaplan 1971 [76] Evans County Heart Study	Early-life: father's occup (7 groups); Early Adult & Adult: occup & social class (5 classes)	None	MI, chronic IHD, AP, sudden death
Gillum 1978 [77] Harvard Alumni Cohort	Early-life: by parental occup (blue/white collar); Adult: assumed to be at least middle class	A confounder summarizing score	AP, HTN, fatal or non-fatal CHD or MI
Burr 1980 [49] South Wales hospital cohort	Father's occup (RG, 3 groups), father unemployed (> than 1 year), family size, current occup (RG, 3 groups)	None	Survived MI
Wadsworth 1985 [72] British 1946 Birth Cohort	Early-life: index of father's occup and parents' edu; Adult: occup (RG), employment & edu for women	Smoking, edu, father's CVD, BMI, more	SBP, DBP
Faresjo 1994 [78] Swedish Study of Men Born in 1913	Early-life: father's SES (3 groups) by occup, social class; Adult: same; all mobility relative to child SES	SBP, cholesterol, smoking	MI
Lynch 1994 [38] Kuopio Study	Early-life: SES index (3 groups); Adult: current income (2 groups)	None	CVD mortality
Blane 1996 [64] Collaborative Study	Early-life: father's occup (RG, 4 groups); Adult: occup (RG, 4 groups)	BMI, cholesterol, DBP, smoking, activity, FEV1	BMI, cholesterol, DBP, smoking, activity, FEV1
Hart 1998 [75] Collaborative Study	Early-life: father's occup (mnl/non-mnl); Labor force entry: occup (mnl/non-mnl); Adult: occup (mnl/non-mnl), area deprivation index	Smoking, DBP, cholesterol, FEV1, angina, ischemia	CVD mortality & risk factors listed
Davey Smith 1998 [44] Collaborative Study	Early-life: father's occup: (RG, mnl/non-mnl); Adult: occup: (RG, mnl/non-mnl)	Smoking, DBP, BMI, area deprivation, more	Mortality from CHD & stroke
Poulton 2002 [73] Dunedin Multidisciplinary Study	Early-life: parental occup (6 groups) at 0, 3, 5, 7, 9, 11, 13 & 15 years; Adult: current occup (3 groups)	Infant health index, gender, adult SES	BMI, WHR, SBP, smoking, alcohol dependence
Pensola 2003 [79] Finnish census cohort	Early-life: father's occup (mnl vs. non-mnl); Adult: current occup (mnl vs. non-mnl)	None	CVD mortality

AP = Angina pectoris; BMI = Body mass index; CHD = Coronary heart disease; DBP = Diastolic blood pressure; Edu = Education; FEV1 = Forced expiratory volume in 1 second; HTN = Hypertension; IHD = Ischemic heart disease; MI = Myocardial infarction; Mnl = Manual occupational class; Non-mnl = Non-manual occupational class; Occup = Occupation; RG = Registrar General's social class categories; SBP = Systolic blood pressure; WHR = Waist-to-hip ratio.

Four studies examined differences in CVD risk between stable low-SES trajectory and stable high-SES trajectory individuals [38,44,75,79]. Three reported that individuals with stable low-SES trajectories had a greater CVD risk than stable high-SES trajectory individuals [44,75,79]; the fourth reported a marginally significantly greater risk [38]. These results are similar to those of most cumulative SES studies (described below) in that greater exposure to low SES was associated with increased CVD risk.

### Social trajectory studies: methodologic issues

In these studies the unit of analysis is a trajectory, permitting the impact of change over time to be examined. However, the socioeconomic trajectories in most reviewed studies were limited to two time points, and groups compared tended to share the same SES at one of these time points. These similarities may partly explain why seven of ten studies did not report an association between social mobility and risk of CVD risk factors or events. Social trajectory studies incorporating SES at three or more time points allow for the analysis of more informative trajectories than studies evaluating SES at only two points [75]. Yet, even with large studies, analysis becomes cumbersome if more than two or three SES levels at three time points are measured. Additionally, the impact of uncommon (e.g. downward) trajectories are difficult to study due to the small numbers of individuals who typically comprise them.

### Cumulative SES studies: overview

These studies tested the operation of the cumulative life course conceptual model, typically by summing the number of times participants experienced unfavorable SES situations during early, middle or later life, and creating SES indices representing the accumulation of these experiences [45,52,53,68,79,81,82]. For example, three of these studies summed the number of times a participant (or their parents during the participant's childhood) had been in a manual occupation to create an index of accumulated low-SES exposure [68,81,82]. Cumulative SES was also measured using indices of occupational class, socioeconomic categories, exposures to negative socioeconomic experiences/conditions, income, and housing conditions. [45,52,53,68,81,82].

### Cumulative SES studies: summary of results

Table 5 summarizes the seven cumulative SES studies (see Additional file 4 for greater detail). All authors reported that participants' cumulative life course exposure to low SES conditions was associated with increases in CVD outcome, supporting the cumulative life course SES hypothesis. Several studies indicated that cumulative SES was a more powerful predictor of CVD morbidity and/or mortality than adult or early-life SES alone [45,53,79,81]. In studies that adjusted for CVD risk factors, graded associations were attenuated but remained strong in two studies [81,82] and were greatly attenuated in another [45].

**Table 5:** Summary of SES – CVD life course studies using a cumulative SES design

**1^st ^Author, year & reference number ** **Study name**	**Cumulative SES measure(s)**	**Variables adjusted for other than age**	**CVD risk factor(s) / outcomes measured**
Davey-Smith 1997 [82] Collaborative Study	Sum of # of times at mnl vs. non-mnl SES using father's, own first, & own current occup class	BMI, DBP, FEV1, cholesterol, smoking, AP, ischemia, more	CVD, mortality, AP, smoking, BMI, DBP, cholesterol, more
Heslop 2001 [81] Collaborative Study	Sum of # of times at mnl vs. non-mnl SES using father's, own first, & own current class	DBP, BMI, FEV1, cholesterol, activity, smoking, alcohol	CVD mortality, DBP, BMI, FEV1, exercise, smoking, alcohol, more
Wamala 2001 [45] Stockholm Study	Sum of # of instances (0–6) of SES disadvantage (large family, born last, low edu, blue-collar/ housewife, economic hardship)	Height, HDL, HTN, marriage, fibrinogen, obesity, smoking, more	Cases: CHD event (acute MI, unstable/ recurrent AP)
Davey-Smith 2002 [52] Collaborative Study	Sum of # of risks (0–6): Father mnl SES, left edu at < 15 years, current mnl SES, smoking, high alcohol, high deprivation area	None	CVD mortality
Lawlor 2002 [68] British Women's Heart Study	Cross-classification of father's longest occup and current occup (RG, mnl/non-mnl)	None	Insulin resistance, HTN, smoking, triglycerides, LDL, HDL, BMI, more
Claussen 2003 [53] Oslo Mortality Study	Early life: Index of housing conditions (scored 0–7) Adulthood: standardized income (7 groups)	None	CVD mortality
Pensola 2003 [79] Finnish census cohort	Sum of # of times in mnl vs. non-mnl class, by father's occup & own occup at 30–34	None	CVD mortality

AP = Angina pectoris; BMI = Body mass index; CHD = Coronary heart disease; DBP = Diastolic blood pressure; Edu = Education; FEV1 = Forced expiratory volume in 1 second; HTN = Hypertension; MI = Myocardial infarction; Mnl = Manual occupational class; Non-mnl = Non-manual occupational class; Occup = Occupation; RG = Registrar General's social class categories.

Davey-Smith et al. (2002) employed a unique cumulative SES measure, combining early and later-life occupational class experience with the CVD risk factors of smoking and heavy alcohol consumption [52]. They reported a marked, graded relationship between the number of negative SES exposures and risk of CVD mortality.

Only a large cohort study of Norwegians reported a statistically significant supra-multiplicative interaction between early- and later-life SES on risk of CVD [53]. Another study reported marginally statistically significant supra-multiplicative interaction effects between early and later life [45].

### Cumulative SES studies: methodologic issues

The reported associations between cumulative SES and CVD risk are more consistent than those reported in the other life course study designs. However, three of the seven studies evaluated were based on the same cohort [52,81,82]. Cumulative life course SES variables include current SES, and therefore may be conflating the effect of current SES with that of SES over the life course. For example, in Pensola et al. (2003), social mobility analyses suggested that the observed gradient in CVD mortality associated with cumulative manual occupational class was driven primarily by the impact of current occupational status. Additionally, cumulative indices implicitly assume that a specific negative life experience or situation has the same impact regardless of when it occurs in an individual's lifetime, with no distinction made between the impact of childhood versus adult events on risk of disease [45]. Furthermore, some cumulative SES studies combined disparate measures into a single, lifetime index variable, or summed the number of times different negative events or exposures occur [45,52]. This approach assumes that different types of exposures have an equivalent impact on the risk of CVD. Evidence supporting these assumptions is not provided in these studies.

## Discussion

### General life course SES study issues

Certain general limitations and assumptions of life course SES studies should be considered. First of all, SES is a theoretical construct operationalized using various measures (e.g., income, occupation, education) that tap into different components of this construct. Despite several proposed SES evaluation schemes [83-85], there is no overarching theory in the biomedical literature providing a rationale for the use of specific SES measures. Occupational status, the SES measure most commonly used in the studies reviewed, is assumed to be an adequate proxy of SES, but in fact represents only one component of SES. As associations of a given SES measure with CVD risk factors are not always consistent with those of other SES measures [47,60], it is important to consider that results may depend upon the proxy SES measure employed [64,81].

Most life course studies used retrospective cohorts or a case-control design, relying on participants' recall of early life SES. There has been little systematic evaluation of the validity of recalled early life circumstances or of the potential for such recall errors to bias associations. In studies directly comparing the impact of childhood and adult SES on adult CVD risk, greater error in childhood (vs. adult) SES measures may underestimate the true impact of child SES [86,87]. Additionally, selection bias due to either loss to follow-up or selective survival may distort findings. Studies using cohorts followed from birth or early life probably do not have these limitations [14,28,47,62,71,72,77,88-93] and may avoid the problem of changing occupational status and workplace functions across time if they establish SES scales at the time of measurement and update them as appropriate. As more studies analyze data from prospective cohorts starting in early life, concerns about unequal measurement error and the changing status of specific occupations should decrease [14,54,73,89,93,94].

Most studies were limited to white males. While the number of studies including women has increased in recent years, minority groups remain underrepresented in most life course studies. These groups may interact differently with the economic and/or social systems of the majority group; there are also established associations between minority status, low SES, and increased CVD risk [5,84,85]. The lack of information on the manner in which life course SES relates to adult CVD risk in minorities should be addressed.

### Support for life course effects on CVD risk

The majority of the early SES → outcome group studies' results describe an inverse association between early life SES and risk of adult CHD, non-fatal MI, and CVD mortality. Similarly most early SES → risk factor studies suggest that low early-life SES negatively impacts levels of adult CVD risk factors. Most studies reported relationships between lower early-life SES and elevated alcohol intake, BMI/WHR, and insulin resistance, as well as decreased leisure physical activity. Together, the early SES → outcome and early SES → risk factor studies support the hypothesis that low early-life SES is inversely associated with adult risk of CVD. However, support for the hypothesis of a "direct effect" of early-life SES on risk of CVD mortality is equivocal as findings were less consistent after risk factor adjustment. Additionally, reported associations between early-life SES and adult health behaviors detract from the evidence supporting a latent effects life course model, as they suggest that CVD risk may be driven by learned behaviors.

The operation of social chains of risk or life trajectories described by a pathway life course model are difficult to observe or test, since almost all early SES → risk factor studies consider only two points during the life course. However, significant associations between early-life SES and the adult risk factors suggest behavioral, psychosocial or environmental links that may be best explained as a pathway effect. As described below, the pathway model will need further examination through the use of series of linked studies.

While nine of 10 social trajectory studies reported some relationship between inter- or intra-generational social mobility and CVD-related outcomes, only four were identified as providing evidence in favor of a social mobility hypothesis. Conversely, most studies comparing stable low- and high-SES trajectory groups reported markedly increased CVD mortality among the low-SES group, with many socially mobile groups having a CVD risk between the stable high and low groups. Determining whether these associations are a function of social mobility, or whether they are due to exposure to low SES at some point in the life course and not to mobility, is problematic. Future studies should focus on differentiating between the influences of cumulative SES and social mobility.

Of the conceptual models discussed, the cumulative life course model was the most consistently supported. However, the weight of these findings is limited as much of the data came from one cohort [57,60,61]. Also, specific methodological concerns (e.g., equal weighting of life course periods, conflation of current and life-course SES) need to be considered when interpreting these findings. Future studies conducted in other cohorts that address these concerns will help clarify the viability of cumulative life course SES hypotheses.

### Directions for future research

Researchers have noted the limitations of life course SES study designs examining only one potential pathway linking SES and CVD. Accordingly, recent studies tend to use a combination of SES measures, CVD risk factor and/or outcomes and life course study designs [40,44,45,52,53,68]. These mixed-design studies allow for a comparison of how well different life course SES conceptual models fit the patterns observed in the same data. Such studies are also less likely to overlook patterns of association in the data than studies which only evaluate one possible relationship between SES and CVD.

As modern life course cohorts are followed from childhood, longer prospective studies or series of cross-sectional studies that evaluate associations between early-life experiences and risk factor levels at several time points may provide the opportunity to observe the operation of pathway life course effects. For example, two studies in the early SES → risk factor group [14,90] compared levels of CVD risk factors at two points in young adulthood (22 and 33 years of age). The authors observed an association between childhood SES and adult obesity, which was smaller for participants at 33 than at 23 years of age [14], suggesting a decreasing impact of early life SES on adult health with increasing age. Such information is only obtained through longer ongoing studies or series of interrelated studies.

Individuals' socioeconomic environments may impact their CVD risk factor levels and CVD risk. Community socioeconomic conditions may influence individuals' ability to engage in leisure physical activity or eat a healthy diet [95-97]. Some life course studies have evaluated the community socioeconomic environment, primarily through the use of indicators of neighborhood deprivation [44,48,54,81,82]. Future studies should likewise consider the effect of physical and psychosocial environmental factors.

Life course SES studies have generally failed to consider the length of exposure to the various socioeconomic conditions measured. As this may influence the impact of negative SES experiences on adult health, future cumulative life course studies may benefit from evaluating the effect of length of exposure into their indices.

## Conclusions

The wide range of populations, analysis designs, exposures, and outcomes used in the life course studies reviewed precludes a simple, quantitative analysis of the impact of life course SES on CVD risk. Nevertheless, the results thus far modestly support the existence of life course SES effects on risk of adult CVD. The cumulative life course model is more consistently supported by extant studies than other models. However, the different methodologic issues of each study design make direct comparisons of the relative support for each conceptual model difficult. Analyses utilizing multiple life course designs within the same study offer the best approach to testing which theories best describe the links between life course SES and CVD risk. The inclusion of minority participants, different SES measures, and data from early and middle adulthood in large, prospective, mixed-design studies will allow more informed and generalizable statements to be made about the impact of life course SES on adult disease risk. While this area of research needs methodologic refinement, it offers a promising and informative perspective from which to understand the development of chronic disease.

## Competing interests

The author(s) declare that they have no competing interests.

## Authors' contributions

R. A. Pollitt conducted the literature search, reviewed and categorized the articles and had primary responsibility for writing the manuscript. K. M. Rose assisted in the categorization and summarization of the papers reviewed, and helped to revise the manuscript in response to the reviewer's comments. All authors participated in interpreting the studies' results and preparing the methodologic criticism, provided input on the various drafts, and read and approved the final manuscript.

## Pre-publication history

The pre-publication history for this paper can be accessed here:



## Supplementary Material

Additional File 1 SES – CVD life course studies using an early SES → outcome design Click here for file

Additional File 2 SES – CVD life course studies using an early SES → risk factor design Click here for file

Additional File 3 SES – CVD life course studies using a social trajectory design Click here for file

Additional File 4 SES – CVD life course studies using a cumulative SES design Click here for file
