# Supplementary material for: Evaluating the evidence for models of life course socioeconomic factors and cardiovascular outcomes: a systematic review
Source: BMC Public Health. 2005 Jan 20;5:7. doi: 10.1186/1471-2458-5-7 (PMC548689; doi:10.1186/1471-2458-5-7)
Supplement: Additional File 1 — SES – CVD life course studies using an early SES → outcome design [file 1471-2458-5-7-S1.doc]

**Additional file 1. SES—CVD life course studies using an early SES 🡪 outcome design**

| **1^st^ Author, year & reference #**  **Study name**  **Study size; % male** | **Study design;**  **age at base-line (years)** | **Early life SES measures** | **Variables adjusted for other than age** | **CVD outcomes evaluated** | **Key findings** |
| --- | --- | --- | --- | --- | --- |
| **Acute/ Survived MI, CVD** |  |  |  |  |  |
| Burr 1980 [49]  South Wales hospital cohort  602; 100% M | Case-Control;  40-69 | Father’s occup (RG, 3 groups), father unemployed (> than 1 year), family size | Current occup (RG, 3 groups) | Survived MI (hospital patients) | In all 3 current SES groups, MI patients came from larger families (p < 0.05) & had higher proportion of fathers unemployed for over a year (p < 0.05). |
| Notkola 1985 [40]  East-West Study  1711; 100% M | Retros-pective cohort;  40-59 | 5-level index using father’s occup & farm size (large/medium farmers, small farmers, landless, craftsmen, others/missing) | Occup (6 groups), cholesterol, smoking, height | MI, IHD without MI | East Finland analysis: RR’s vs. men born to large/medium farmers: small farmers: 1.24, landless: 1.63, craftsmen: 0.95, others/missing: 0.73 (no tests for statistical significance conducted). Fully adjusted RR’s: small farmers: 1.22, landless: 1.31, craftsmen: 1.14, others/missing: 0.62. |
| Coggon 1990 [37]  Stoke-on-Trent & Newcastle study  351; 74% M | Case-control;  25-64 | Father’s occup (RG, 5 groups), height, perinatal sibling death | Current occup (RG, 5 groups), smoking | Acute MI | Somewhat elevated risk of MI for lower childhood SES; adjustment had little effect. Adjusted risk of MI vs. birth classes I-III non-manual: III manual: 1.9 (95% CI: 0.6-6.5), IV, V: 2.0 (95% CI: 0.5-7.6), military: 3.9 (95% CI: 0.8-19.2). |
| Hasle 1990 [48]  Danish worker’s union study  1673; 100% M | Nested case-control;  50-67 | 8 variables (yes/no) on parent’s occup, health, household condition, urban residence, edu, illness | None; cases were disability pensioners; controls were union members | Past MI (self-reported) | No significant differences between cases & controls. For entire group: OR for MI, less vs. more school: 1.95 (95% CI: 0.99-3.85), urban residence: 1.39 (95% CI: 0.98-1.98), broken home 1.38 (95% CI: 0.89-2.12). Among controls, broken homes & urban residence associated (p < 0.05) with MI. |
| Kaplan 1990 [43]  Kuopio Study  2679; 100% M | Retros-pective cohort;  42-60 | Factor analysis of edu, occup, farm (yes/no), farm size, perceived wealth | Physical and behavioral CVD risk factors[[1]](#footnote-2) | IHD (max exercise test, EKG) | Childhood SES associated (p < 0.05) with IHD. Adjustment for adult RF’s did not attenuate association, but adjustment for adult SES attenuated association to non-significance. Age-adjusted RR’s vs. High child SES: Med SES 1.35 (95% CI: 1.12-1.64), Low 1.44 (95% CI: 1.17-1.78). RR’s adjusted for adult SES: Med: 1.20 (95% CI: 0.98-1.45), Low: 1.21 (95% CI: 0.97-1.51). |
| Lundberg 1993 [51]  Swedish population cohort study  4216; 49% M | Pros-pective cohort;  30-75 | 4 yes/no variables: economic hardship, large family, broken family, dissension in family | Gender, father’s social class (8 groups) | AP, MI, HTN, weak heart, dizziness | Adjusted OR for childhood variables (for yes vs. no answer): economic hardship: 1.43 (p = 0.003), large family: 1.39 (p = 0.004), broken family: 1.64 (p = 0.001), family dissension: 2.42 (p = 0.001). Entering childhood variables into model together attenuated effect of economic hardship to non-significance. |
| Gliksman 1995 [39]  Nurses’ Health Study  117, 006; 0% M | Pros-pective cohort;  30-55 | Father’s occup (white, blue-collar, farmer, deceased) at 16 years | Husband’s edu, wide range of covariates[[2]](#footnote-3) | Non-fatal MI | Increased risk of MI for women with fathers who were blue-collar: RR 1.23 (95% CI: 1.06-1.42) or deceased: RR 1.35 (95% CI: 1.08-1.69) versus white-collar, when women were 16. Adjustment for all RF’s attenuated RR’s to marginal statistical significance. Women with fathers who were farmers had lower RR: RR 0.93 (95% CI: 0.72-1.19); adjustment led to increased RR. |
| Lamont 2000 [41]  Newcastle 1,000 Families Cohort 347; 44% M | Retros-pective cohort;  49-51 | Birth: father's occup (RG, 4 groups)  5 & 10 years: wage earner’s occup, housing index,[[3]](#footnote-4) # of adverse life events in childhood[[4]](#footnote-5) | Adult SES: occup (RG, 4 groups), smoking, alcohol, diet, SBP, cholesterol, WHR, LDL, HDL, fibrinogen, insulin, more | % variance explained,carotid IMT | Independent contribution of early life was small (3.2% of variance (95% CI: 0.27-6.78) in M, 2.22% (95% CI: 0.21-5.04) in F) compared to adult biological risk markers (9.49% of variance (95% CI: 2.38-14.21) in M, 4.87% (95% CI: 1.56-7.39) in F). Higher total contribution of early life effects, including adult-mediated effects of childhood: 9.15% (95% CI: 2.42-12.26) for M, 4.73% (95% CI: 2.12-6.68) for F. |
| Marmot 2001 [46]  Whitehall II Study  10,308; 67% M | Pros-pective cohort;  35-55 | Childhood: father’s occup (RG, 4 groups), age leaving full time edu;  Labor force entry: occup by civil service grade (high/middle/low) | Adult social class by civil service grade (high/ middle/low), father’s SES, labor force entry SES | Self-reported CHD | Labor force entry & current occup grade both associated with CHD risk, but not childhood SES (unadjusted OR are 1.57 (95% CI: 0.9-2.7), 1.57 (95% CI: 1.0-2.4) and 1.10 (95% CI: 0.7-1.8), respectively). Adjustment for SES at other life course points attenuated all associations. |
| Wamala 2001 [45]  Stockholm Study  584; 0% M | Case-control;  30-65 | Early-life SES disadvantage index (0-3) of 3 variables: large family size, being born last, & low edu | Marital status, adult SES disadvantage, smoking, physical activity, abdominal obesity, HDL, triglycerides, HTN, fibrinogen | Acute MI, or recurrent/ unstable AP | Early life SES disadvantage somewhat associated with increased CHD risk (3 instances of disadvantage vs. none: OR = 2.65, (95% CI: 1.12-6.54); 2 instances: 1.73 (95% CI: 0.96-3.88)). After adjustment, OR for 3 instances: 2.48 (95% CI: 0.90-6.83); 2 instances: 1.34 (95% CI: 0.62-2.88). Later life SES disadvantage had greater effect on CHD risk. |
| Stroke |  |  |  |  |  |
| Gliksman 1995 [39]  Nurses’ Health Study  117, 006; 0% M | Pros-pective cohort;  30-55 | Father’s occup (white-, blue-collar, farmer, deceased) at 16 years | Husband’s edu, wide range of covariates[[5]](#footnote-6) | Stroke (fatal & non-fatal) | Women with deceased fathers when they were 16 had slightly elevated unadjusted stroke risk vs. women of white-collar fathers (RR 1.17 (95% CI: 0.91-1.47)). Women of fathers who were farmers had slightly decreased risk (RR 0.96 (95% CI: 0.74-1.23)). |
| Coggon 1990 [37]  Stoke-on-Trent & Newcastle study  351; 0% M | Case-control;  > 65 | Father’s occup (RG, 5 groups), height, perinatal sibling death | Current occup (RG, 5 groups), smoking | Recent hemis-pheric stroke | Slight or no elevated risk of stroke for those of lower childhood SES. Adjusted risk vs. birth classes I-IIINM: IIIM: 1.5 (0.4-6.3), IV, V: 0.9 (95% CI: 0.2-3.3), father in armed forces: 0.3 (95% CI: 0.0-3.3). |
| Davey Smith 1998 [44]  Collaborative Study  5645; 100% M | Pros-pective cohort;  35-64 | Father's occup (RG, 4 groups), also divided into mnl vs. non-mnl groups | Adult class (6 groups), CVD RF’s,[[6]](#footnote-7) area deprivation, car ownership | Stroke mortality | Lower vs. high father’s SES associated with elevated stroke mortality in unadjusted analysis (p trend = 0.031). Fully adjusted RR of stroke mortality for mnl vs. non-mnl father’s SES: 1.74 (95% CI: 1.05-2.90). |
| Frankel 1999 [54]  Boyd Orr Cohort 3750; 50% M | Retros-pective cohort;  < 20 | Father’s occup (RG, 4 groups + unemployed) | Adult Townsend area deprivation score[[7]](#footnote-8) | Stroke mortality | Linear trend of increasing stroke mortality with decreasing childhood SES (p = 0.01). Adjustment for Townsend deprivation score did not alter findings. |
| Dedman 2001 [56]  Boyd Orr Cohort  4168; 50% M | Retros-pective cohort;  < 20 | Persons/room (crowding), tap water yes/no, toilet type (3 groups), ventilation (3 levels), cleanliness (3 levels) | Childhood SES by father’s occup (by RG), per capita food expenditure & household per capita income; adult SES by Townsend area deprivation score^7^ | Stroke mortality | Poorer housing conditions in childhood were not a major determinant of stroke mortality, although poorer ventilation was associated with higher stroke mortality. For higher crowding, p trend for HR = 0.53, no vs. yes tap water: p trend = 0.53, worse toilet type: p trend = 0.67, worse ventilation: p trend = 0.08, poor cleanliness: p trend = 0.07. Adjustment had minor impact. |
| **CHD Mortality** |  |  |  |  |  |
| Notkola 1985 [40]  East-West Study 1711; 100% M | Retros-pective cohort;  40-59 | 5-level index using father’s occup & farm size (large/medium farmers, small farmers, landless, craftsmen, others/missing) | Occup (6 groups), cholesterol, smoking, height | CHD mortality | East Finland analysis: Men born to small farmers and landless fathers had elevated risk of CHD death. RR’s vs. men born to large/medium farmers: small farmers: 1.44, landless: 1.88, craftsmen: 0.69, others/missing: 1.17 (no tests for statistical significance conducted). |
| Lynch 1994 [38]  Kuopio Study  2636; 100% M | Pros-pective cohort;  42-60 | SES index (3 groups), by parents’ edu, occup, farm yes/no & size, perceived wealth | Adult SES by current income (2 groups) | CVD mortality | Childhood SES not clearly associated with CVD mortality. Low adult income vs. high adult income associated with CVD mortality (RR 2.37 (95% CI: 1.51-3.70)). |
| Vagero 1994 [50]  Uppsala Birth Cohort Study  404,450; 100% M | Retros-pective cohort;  25-40 | Occup of head of household (mnl, non-mnl, unemployed) | Own occup (mnl, non-mnl, unemployed) | IHD mortality | Indication of independent effect of child SES on IHD mortality risk. Unadjusted RR vs. non-mnl child SES: mnl: 2.29 (95% CI: 1.51-3.46), unemployed: 2.23 (95% CI: 1.08-4.59). Adjusted RR, mnl: 1.99 (95% CI: 1.30-3.05), unemployed: 1.82 (95% CI: 0.88-3.77). |
| Gliksman 1995 [39]  Nurses’ Health Study  117, 006; 0% M | Pros-pective cohort;  30-55 | Father’s occup (white-, blue-collar, farmer, deceased) at 16 years | Husband’s edu, wide range of covariates[[8]](#footnote-9) | CVD mortality | RR vs. women of white-collar fathers: blue-collar father: 1.09 (95% CI: 0.86-1.37), farmer: 0.69 (95% CI: 0.46-1.05), father deceased: 1.20 (95% CI: 0.84-1.72). After adjustment, increased risk of children of blue-collar and deceased fathers disappeared; somewhat decreased risk of women of farmers remained. |
| Davey Smith 1998 [44]  Collaborative Study  5645; 100% M | Pros-pective cohort;  35-64 | Father's occup (RG, 4 groups), also divided into mnl vs. non-mnl groups | Adult class (RG, 6 groups), CVD RF’s,[[9]](#footnote-10) area deprivation, car ownership | CHD mortality | Lower vs. high father’s SES associated with elevated CHD mortality in unadjusted analysis (p trend 0.0003). Fully adjusted RR of CHD mortality for mnl vs. non-mnl fathers’ SES: 1.26 (95% CI: 1.01-1.58). |
| Hart 1998 [42]  Collaborative Study  5567; 100% M | Pros-pective cohort;  35-64 | Early SES: father’s occup  Labor force entry: occup  SES at screening: occup (All 3 time points used RG, 4 groups) | None; Relative Index of Inequality (RII) used to compare groups | CVD mortality | Increasing absolute risk of CVD mortality as childhood SES decreases (p trend < 0.0001); Relative Index of Inequality (RII) = 1.68 (95% CI: 1.30-2.17). Similar findings for labor force entry SES (p trend < 0.0001, RII = 1.62 (95% CI: 1.26-2.09)) and for SES at screening (p trend < 0.0001, RII = 1.79 (95% CI: 1.40-2.29)). |
| Frankel 1999 [54]  Boyd Orr Cohort  3750; 50% M | Retros-pective cohort;  < 20 | Father’s occup (RG, 4 groups + unemployed) | Adult Townsend area deprivation score[[10]](#footnote-11) | CHD mortality | Linear trend of increasing CHD mortality with decreasing childhood SES (p = 0.12). Adjustment for Townsend deprivation score did not alter findings. |
| Davey Smith 2001 [47]  Glasgow Alumni Cohort  8396; 100% M | Pros-pective cohort;  univer-sity age | Childhood SES by father’s social class, (RG, 5 groups) | SBP, smoking (most had privileged adult social environment) | CVD mortality | Strong association between childhood SES & CVD mortality. RR vs. RG class I: class II: 1.51 (95% CI: 1.08-2.11), III: 1.63 (95% CI: 1.17-2.27), IV: 1.85 (95% CI: 1.12-3.07), V: 2.36 (95% CI: 1.11-4.99). Trend of increasing CVD mortality risk with decreasing early SES (p trend = 0.002). Adjustment for SBP & smoking had little effect. |
| Dedman 2001 [56]  Boyd Orr Cohort 4168; 50% M | Retros-pective cohort;  < 20 | Persons/room (crowding), tap water (yes/no), toilet type (3 groups), ventilation (3 levels), cleanliness (3 levels) | Childhood SES by father’s occup (by RG) & per capita food expenditure & household per capita income; adult SES by Townsend area deprivation score^10^ | CHD mortality | Poorer childhood housing conditions were in general associated with, but were not a major determinant of, CHD mortality. For higher crowding, p trend for HR = 0.11; no vs. yes tap water: p trend = 0.03; worse toilet type: p trend = 0.13, worse ventilation: p trend = 0.14, worse cleanliness: p trend = 0.37. Adjustment had small effect. |
| Davey Smith 2002 [52]  Collaborative Study  5628; 100% M | Pros-pective cohort;  35-64 | Father’s occup (mnl/non-mnl) | Occup (mnl/non-mnl), alcohol, smoking, area deprivation, age at leaving edu | CVD mortality | Mnl vs. non-mnl unadjusted RR for CVD mortality: 1.61 (95% CI: 1.39-1.88). Stratification by each adult risk factor did not attenuate the association between childhood SES and CVD mortality below statistical significance (p < 0.05). |
| Claussen 2003 [53]  Oslo Mortality Study  101,487; 50% M | Retros-pective cohort;  31-50 | Index of housing conditions items;[[11]](#footnote-12) Relative Index of Inequality (RII) used to compare groups | Adult household income (7 groups by income); RII used to compare groups | CVD mortality | CVD mortality more strongly related to childhood than adult SES for both M & F. RII for childhood SES: M: 2.79 (95% CI: 1.71-4.55), F: 3.96 (95% CI: 1.52-10.3). After adjusted for adult SES, RII for M: 2.68 (95% CI: 1.64-4.38), F: 3.80 (95% CI: 1.45-9.96). Supra-multiplicative interaction (p < 0.05) observed between childhood & adult SES. |
| Osler 2003 [55]  Project Metropolit  7493; 100% M | Pros-pective cohort;  49 | Father’s social class (3 groups: high/ middle, working, unknown) by occup status | Birth weight, cognitive function (IQ score) at age 12 | CVD mortality | Men with working class & unknown class fathers had higher HR for CVD death than men with high/middle class fathers (p < 0.05). Adjustment attenuated HR (p > 0.05 for men with working class fathers). |

AP = Angina pectoris; BMI = Body mass index; CHD = Coronary heart disease; CVD = Cardiovascular disease; DBP = Diastolic blood pressure; Edu = Education; F = Female; FEV1 = Forced expiratory volume in 1 second; HR = Hazard ratio; HTN = Hypertension; IHD = Ischemic heart disease; M = Male; MI = Myocardial infarction; Mnl = Manual occupational class; Non-mnl = Non-manual occupational class; Occup = Occupation; OR = Odds ratio; RF = Risk factor; RG = Registrar General’s social class categories; RR = Relative risk; SBP = Systolic blood pressure; SES = Socioeconomic status; WHR = Waist-to-hip ratio.

1. Adult SES (summary index), smoking, HDL, LDL, fibrinogen, selenium, age, height, diagnoses of IHD and HTN, CVD meds. [↑](#footnote-ref-2)
2. Adjusts for HTN, cholesterol, diabetes, BMI, adult SES, smoking, family & personal med history, meds use, exercise, alcohol, diet, birth weight, and breastfeeding. [↑](#footnote-ref-3)
3. Housing conditions at birth and in childhood scored for presence of up to 3 or more of: lack of hot water, shared toilet, overcrowding, and dampness or poor repair. [↑](#footnote-ref-4)
4. Scored for presence of up to 2 or more of: parental divorce or separation, death of a parent, parental incapacity due to illness, serious debt, and parental criminal activity or cruelty. [↑](#footnote-ref-5)
5. Adjusts for HTN, cholesterol, diabetes, BMI, adult SES, smoking, family & personal med history, meds use, exercise, alcohol, diet, birth weight, and breastfeeding. [↑](#footnote-ref-6)
6. CVD risk factors: smoking, DBP, cholesterol, BMI, and FEV1 score. [↑](#footnote-ref-7)
7. Constructed from census data on levels of unemployment, overcrowding housing, housing tenure and car ownership in the Health Authority of residence. [↑](#footnote-ref-8)
8. Adjusts for HTN, cholesterol, diabetes, BMI, adult SES, smoking, family & personal medical history, meds use, exercise, alcohol, diet, birth weight, and breastfeeding. [↑](#footnote-ref-9)
9. Adjusts for social class, area deprivation, car ownership, smoking, DBP, cholesterol, BMI, and FEV1. [↑](#footnote-ref-10)
10. Constructed from census data on levels of unemployment, overcrowding housing, housing tenure and car ownership in the Health Authority of residence. [↑](#footnote-ref-11)
11. Housing Index was from 1 (poor) to 7 (well off), and used the following items: dwelling type, # rooms, ownership, telephone, toilet, bath. [↑](#footnote-ref-12)
