# Supplementary material for: Evaluating the evidence for models of life course socioeconomic factors and cardiovascular outcomes: a systematic review
Source: BMC Public Health. 2005 Jan 20;5:7. doi: 10.1186/1471-2458-5-7 (PMC548689; doi:10.1186/1471-2458-5-7)
Supplement: Additional File 2 — SES – CVD life course studies using an early SES → risk factor design [file 1471-2458-5-7-S2.doc]

**Additional file 2. SES—CVD life course studies using an early SES 🡪 risk factor design**

| **1^st^ Author, year & reference number**  **Study name**  **Study size;**  **% male** | **Study design;**  **age at baseline (years)** | **Early life SES measures** | **Adult SES measures** | **Variables adjusted for other than age** | **CVD risk factor(s) measured** | **Key findings** |
| --- | --- | --- | --- | --- | --- | --- |
| Arnesen 1985 [65]  Tromso Heart Study  14,652; 51% M | Cross-sectional;  20-54 | 4-level index of household economic conditions (very difficult to very good) | None | Alcohol, BMI, physical activity, smoking | Cholesterol, SBP, DBP, glucose, height, weight, BMI, smoking | Very difficult vs. very good childhood conditions assoc (p < 0.05) with cholesterol, height (inversely), & % male smokers. P trend < 0.05 for height, and for cholesterol in women, after covariate adjustment. |
| Notkola 1985 [40]  East-West Study  1711; 100% M | Retros-pective cohort;  40-59 | 5-level index using father’s occup & farm size (large/medium farmers, small farmers, landless, craftsmen, others/missing) | Occup (6 groups) | None | Smoking, cholesterol, SBP | East Finland analysis: Childhood SES not associated with adult cholesterol, inversely associated with smoking and SBP (p > 0.05) |
| Wadsworth 1985 [98]  Medical Research Council National Survey of Health Study  3322; 62% M | Pros-pective cohort;  36 | Index of father’s occup (RG, mnl/non-mnl) and parents’ edu | Occup (RG) & employment status (& edu for women) | Smoking, father’s HTN or IHD death, BMI, birth weight, adult edu | SBP, DBP | Childhood in lowest social group associated with higher SBP and DBP compared to highest child social group for M (p < 0.01) and F (p < 0.05). Inclusion of these variables in a multivariate model resulted in elimination of their predictive value. |
| Braddon 1986 [71] British 1946 Birth Cohort  3322; 62% M | Pros-pective cohort;  36 | 2 social class indices of father’s occup: 1) mnl/non-mnl, and 2) high non-mnl, low non-mnl, high mnl, low mnl | Occup (RG, 8 groups), edu (high/low) | Smoking, relative weight at 11, adult edu, BMI at 26, marital status, parity | Obesity (BMI > 30.0 for men, > 29.1 for women) | Manual group in childhood associated with obesity (p < 0.001). For men but not women, adjustment for relative weight at age 11 & edu reduced association to non-significance. Obesity also associated with edu and adult SES. |
| Peck 1994 [66]  Swedish census cohort study  12695; 50% M | Pros-pective cohort;  16-74 | Father’s occup (7 groups by Statistics Sweden classification) | Occup (7 groups, Statistics Sweden classification) | None | Smoking, no leisure physical activity | Lower childhood SES associated (p < 0.10) with increased adult smoking and decreased adult leisure physical activity for both M & F. |
| Blane 1996 [64]  Collaborative Study  5645; 100% M | Cross-sectional;  35-64 | Father’s occup (RG, 4 groups) | Occup (RG, 4 groups) | Adult SES | DBP, cholesterol, physical activity, smoking, BMI, FEV1 | Father’s & own SES associated (p < 0.05) with all RF’s except: BMI not with current SES; smoking & exercise not with father’s SES. Suggestion of gradient in RF’s for both father’s & own SES. Regression coefficients of adult SES were larger than those of childhood SES for all variables except BMI. |
| Lynch 1997 [99]  Kuopio Study  2674; 100% M | Pros-pective cohort;  42-60 | Child: Index (3 groups) based on: parents’ edu, occup, perceived wealth, farm ownership & size  Adolescence: edu (3 groups) | Occup, income, housing, job security, work injury/ disability, possessions | Energy intake (for diet measure) | Smoking, frequency drunk, physical activity, obesity, diet | Graded association between SES at all 3 time points & obesity, adult behaviors. Several adult risk factors associated with lower childhood SES (p < 0.05 for physical activity, diet, frequency drunk for poor and/or middle child SES vs. high child SES). |
| Power 1997a [14]  1958 British Birth Cohort  11407; 49% M | Pros-pective cohort;  33 | Child: Father’s occup (RG, 4 groups)  At 23 years: Occup (RG, 4 groups), edu (5 groups) | At 33 years: Occup (RG, 4 groups) | None | BMI (obesity) | Slope of inequality[[1]](#footnote-2) by birthweight SES for obesity[[2]](#footnote-3) at 23 years: M: 4.80 (95% CI: 2.09-11.00), F: 2.84 (95% CI: 1.62-4.99); at 33 years: M: 2.19 (95% CI: 1.51-3.19), F: 1.99 (95% CI: 1.46-2.72). BMI inequality was reduced from 23 to 33, but obesity prevalence increased. |
| Power 1997b [90]  1958 British Birth Cohort  11407; 49% M | Pros-pective cohort;  33 | Child: Father’s occup (RG, 4 groups)  At 23 years: Occup (RG, 4 groups), edu (5 groups) | At 33 years: Occup (RG, 4 groups) | None | Smoking, BMI (obesity) | Birth SES associated with % smokers at 23-33 years and % obese^2^ at 33 (p trend < 0.001). Edu at 23 associated with obesity at 33 (p trend < 0.01). |
| Davey Smith 1998 [44]  Collaborative Study  5645; 100% M | Pros-pective cohort;  35-64 | Father's occup (4 groups) | Occup (6 groups) | None | Smoking, DBP, cholesterol, BMI, FEV1 | Inverse association between child SES & all adult CVD RF’s measured except cholesterol, to which child SES had a positive association (p for trend = 0.0001 for all). |
| van de Mheen 1998 [63]  Longitudinal Study, Netherlands  13854; 49% M | Pros-pective cohort;  25-74 | Father’s occup (6 groups, using Erikson, Goldthorpe, and Portacarero scheme) | Occup (6 groups, using Erikson, Goldthorpe, and Portacarero scheme) | Adult SES | BMI, smoking, alcohol, leisure physical activity | Child SES inversely associated with BMI > 27, excessive alcohol consumption, and smoking (p trend < 0.01), and no leisure physical activity (p trend < 0.05). Association remained significant for BMI, alcohol use, and smoking after adjustment for current SES. |
| Brunner 1999 [67]  Whitehall II Study  6980; 68% M | Cross-sectional;  35-55 | Father’s occup (RG, 4 groups) | Occup (Civil Service grade, 4 groups) | Adult SES | Smoking, physical activity, WHR, HDL cholesterol, triglycerides, fibrinogen, glucose, BMI | Lowest childhood SES vs. highest associated with physical activity, smoking, HDL & overweight in M & F (p trend < 0.05), & with WHR, cholesterol, triglycerides & fibrinogen in F. The combination of low childhood & current SES strongly associated with higher BMI (p < 0.001). |
| Davey Smith 2002 [52]  Collaborative Study  5628; 100% M | Pros-pective cohort;  35-64 | Father's occup (mnl/non-mnl) | Occup (mnl/non-mnl) | None | Smoking, alcohol, area deprivation | Manual childhood SES associated (p < 0.05) with smoking, high alcohol consumption, higher area deprivation, and manual adult SES. |
| Lawlor 2002 [68]  British Women’s Heart Study  4286; 0% M | Cross-sectional;  60-79 | Father’s occup (RG, 6 groups) | Current occup (RG, 6 groups) | Adult SES | Insulin resistance, SBP, cholesterol, BMI, WHR, smoking, triglycerides, heavy alcohol intake | Lower child & adult SES each associated (p < 0.05) with insulin resistance, BMI, smoking, triglycerides & alcohol intake. Association with insulin resistance was stronger for childhood class than for adult class, and unaffected by adjustment for adult class. |
| Poulton 2002 [73]  Dunedin Multidisciplinary Study  1000; 52% M | Pros-pective cohort;  birth | Average of highest parental occup (6 groups based on New Zealand census data) assessed at birth & 3, 5, 7, 9, 11, 13 & 15 years (scores then grouped high/medium/low) | Current occup (age 26) (based on New Zealand census data) grouped high/medium/low | Infant health index,[[3]](#footnote-4) gender, adult SES | BMI, WHR, SBP, cardio-respiratory fitness (VO_2_max/kg), smoking, alcohol dependence | After adjustment, participants with low-SES homes had higher BMI (p = 0.003), WHR (p = 0.001), SBP (p = 0.03) and alcohol dependence (p < 0.05), and worse cardio-respiratory fitness (p = 0.009) than those from high-SES homes. |
| Lawlor 2003 [69]  British Women’s Heart Study  1394; 0% M | Cross-sectional;  60-79 | Childhood: Father’s longest held occup (mnl vs. non-mnl) | Longest held occup (husband’s occup if married) (RG, 6 groups) | Smoking, adult SES, obesity, birth weight, offspring birth weight, leg length | HOMA, SBP, HDL, triglycerides | Mnl vs. non-mnl childhood SES associated (p < 0.05) with insulin resistance, SBP, triglycerides levels, & lower HDL. Adjustment for obesity, smoking, & early life factors attenuated assoc, to non-significance, except for association with HDL. |
| Parker 2003 [70]  Newcastle 1000 Families Study  358; 43 M% | Pros-pective cohort;  49-51 | Birth: Father’s occup (RG, 4 groups) & housing conditions;[[4]](#footnote-5)  5 & 10 years: Wage earner’s occup, housing conditions, adverse life events[[5]](#footnote-6) | Occup of wage earner (RG, 4 groups) | None | CMS, [[6]](#footnote-7) BMI, WHR, fasting insulin, triglycerides, HDL | Birth SES associated (p < 0.05) with adult BMI, WHR in M & with triglycerides in F. Adverse child events associated (p < 0.05) with WHR in M & with WHR & insulin in F. Early life socioeconomic variables unrelated to CMS scores. 2/3 of explained CMS score variation in F & almost 1/2 in M accounted for by adult RF’s. |

BMI = Body mass index; CMS = Central metabolic syndrome; CVD = Cardiovascular disease; DBP = Diastolic blood pressure; Edu = Education; F = Female; FEV1 = Forced expiratory volume in 1 second; HOMA = Homeostasis model assessment score; HR = Hazard ratio; HTN = Hypertension; M = Male; MI = Myocardial infarction; Mnl = Manual occupational class; Non-mnl = Non-manual occupational class; Occup = Occupation; RF = Risk factor; RG = Registrar General’s social class categories; SBP = Systolic blood pressure; SES = Socioeconomic status; WHR = Waist-to-hip ratio.

1. Indicates proportional increase in odds for the bottom vs. the top of the social hierarchy. [↑](#footnote-ref-2)
2. Obesity evaluated by BMI > 30 for M, > 28.6 for F. [↑](#footnote-ref-3)
3. Infant health index composed of sum of # of complications including maternal diabetes, glycosuria, epilepsy, HTN, eclampsia, antepartum hemorrhage, accidental hemorrhage, placenta praevia, previous small baby, higher-risk gestational age, higher-risk birthweight, more. [↑](#footnote-ref-4)
4. Housing conditions at birth and in childhood scored for presence of up to 3 or more of: lack of hot water, shared toilet, overcrowding, and dampness or poor repair. [↑](#footnote-ref-5)
5. Two or more of the following during childhood: parental separation, death, incapacity, debt; criminality/ cruelty. [↑](#footnote-ref-6)
6. Central Metabolic Syndrome determined by principal components analysis using: BMI, WHR, SBP, DBP, HDL, triglycerides, fasting & 2-hour post challenge glucose & insulin. [↑](#footnote-ref-7)
