# Supplementary material for: Evaluating the evidence for models of life course socioeconomic factors and cardiovascular outcomes: a systematic review
Source: BMC Public Health. 2005 Jan 20;5:7. doi: 10.1186/1471-2458-5-7 (PMC548689; doi:10.1186/1471-2458-5-7)
Supplement: Additional File 3 — SES – CVD life course studies using a social trajectory design [file 1471-2458-5-7-S3.doc]

**Additional file 3. SES—CVD life course studies using a social trajectory design**

| **1^st^ Author, year & reference #**  **Study name**  **Study size;**  **% male** | **Study design;**  **age at baseline (years)** | **SES measures** | **Variables adjusted for other than age** | **CVD risk factor(s) / outcomes measured** | **Key findings**  **Inter = Inter-generational mobility**  **Intra = Intra-generational mobility** |
| --- | --- | --- | --- | --- | --- |
| Kaplan 1971 [76]  Evans County Study  2850; 100% M | Cross-sectional;  22 & older | Childhood: Father's occup (Hollingshead scale, 7 groups);  Early Adulthood & Adulthood: Occup & social class (McGuire-White scale, 5 classes) | None | MI, chronic IHD, AP, sudden death | Inter & Intra: UM protective in upper-class men vs. stable upper-class men, but led to some CHD excess in lower-class men vs. stable lower-class men. No confidence intervals or significance tests. |
| Gillum 1978 [77]  Harvard Alumni Cohort  13,728; 100% M | Pros-pective cohort;  15-29 | Childhood: By parental occup (blue/white collar);  Adulthood: All assumed to be at least middle class as Harvard graduates | A confounder summarizing score[[1]](#footnote-2) | AP, HTN, fatal or non-fatal CHD or MI | Inter: UM by occup associated with increased combined CHD risk vs. stable high occup (p < 0.05). There was no stable low group. The association was slightly attenuated by adjustment, but remained significant (p < 0.05). |
| Burr 1980 [49]  South Wales hospital cohort  602; 100% M | Case-control;  40-69 | Father’s occup (RG, 3 groups), father unemployed (> than 1 year), family size, current occup (RG, 3 groups) | None | Survived MI (hospital patients) | Inter: No clear evidence that the MI cases had a greater rise in social status from childhood to adulthood than the controls. |
| Wadsworth 1985 [72]  British 1946 Birth Cohort  3322; 62% M | Pros-pective cohort;  36 | Childhood: Index of father’s occup (mnl/non-mnl) and parents’ edu; Adulthood: Occup (RG), & employment status & edu for women | Smoking, father’s HTN/IHD death, BMI, birth weight, edu | SBP, DBP | Inter: Men: UM via university edu associated with decreased SBP vs. stable low-SES group (p < 0.01). UM *within* manual classes associated with elevated SBP (p < 0.05). Women: no social mobility effects observed. |
| Faresjo 1994 [78]  Swedish Study of Men Born in 1913  682; 100% M | Pros-pective cohort;  60 | Childhood: Father’s SES (3 groups) by occup & social position (Swedish social group scale);  Adulthood: Same scale; mobility was only measured relative to childhood SES (e.g., upwardly mobile/stable/downwardly mobile) | SBP, cholesterol, smoking | MI | Inter: Relatively DM had slightly elevated MI risk vs. stable & relatively UM groups (p = 0.1072). Adjustment for RF’s attenuated this relationship. Relatively UM had slightly reduced MI risk vs. stable group. |
| Lynch 1994 [38]  Kuopio Study  2636; 100% M | Pros-pective cohort;  42-60 | Childhood: SES index (3 groups), by parents’ edu, occup, perceived as wealthy, farm yes/no & size;  Adulthood: Current income (2 groups) | None | CVD mortality | Inter: UM men had similar CVD risk as stable, high-SES men, who had less risk (RR 2.02 (95% CI: 0.9-4.54)) than stable low-SES men. DM men had risk in between the stable low and stable high groups. |
| Blane 1996 [64]  Collaborative Study  5645; 100% M | Cross-sectional;  35-64 | Childhood: Father’s occup (RG, 4 groups);  Adulthood: Occup (RG, 4 groups) | BMI, cholesterol, DBP, smoking, activity, FEV1 | BMI, cholesterol, DBP, smoking, physical activity, FEV1 | Inter: Tendency for increase in levels of CVD RF’s with more exposure to lower SES levels; no analysis provided. |
| Hart 1998 [75]  Collaborative Study  5567; 100% M | Pros-pective cohort;  35-64 | Early SES: Father’s occup (mnl/non-mnl);  Labor force entry: Occup (mnl/non-mnl);  Adulthood: Occup (mnl/non-mnl), area deprivation index;  3 mobility trajectories examined: Early SES to adulthood, early SES to labor entry, and labor entry to adulthood | Smoking, DBP, cholesterol, FEV1, angina, car ownership, ischemia | CVD mortality & risk factors at left | Inter & Intra: In each of the 3 trajectories, socially mobile men had risks & RF levels between low stable & high stable SES men. Stable non-mnl group had lower risk than stable mnl group for all 3 trajectories (p < 0.05). |
| Davey Smith 1998 [44]  Collaborative Study  5645 ; 100% M | Pros-pective cohort;  35-64 | Childhood: Father's occup: (RG, mnl/non-mnl);  Adulthood: Occup: (RG, mnl/non-mnl) | Smoking, DBP, car ownership, cholesterol, BMI, FEV1, area deprivation | Mortality from CHD & stroke | Inter: UM had slightly reduced mortality rate vs. stable manual; DM had increased rate for CHD vs. stable non-mnl (p < 0.05). Stable non-mnl had lower rate than stable mnl (p < 0.05). Mobile groups had rates between stable low & stable high groups. |
| Poulton 2002 [73]  Dunedin Multi-disciplinary Study  1000; 52% M | Pros-pective cohort;  Birth | Childhood: Average of highest occup of either parent (6 groups based on New Zealand census data) assessed at birth and 3, 5, 7, 9, 11, 13 and 15 years of age (scores then grouped high/medium/low); only those with low or high childhood SES used in mobility analysis;  26 years: Current occup (based on New Zealand census data; grouped high/medium/low) | Infant health index,[[2]](#footnote-3) gender, adult SES | BMI, WHR, SBP, smoking, cardiorespiratory fitness, alcohol abuse | Inter: UM had lower WHR (p = 0.03) than stable-high SES group, whereas DM had worse cardio-respiratory fitness than stable-high group (p = 0.04). UM and DM groups had RF levels in between stable low and stable high SES trajectory groups for BMI, WHR, fitness, and smoking. |
| Pensola 2003 [79]  Finnish census cohort  112,735; 100% M | Retros-pective cohort;  30-42 | Childhood: Father’s occup (mnl vs. non-mnl);  Adulthood: Current occup (mnl vs. non-mnl); farmers & entrepreneurs excluded at both time points | None | CVD mortality | Inter: UM men had similar CVD mortality rate to stable non-mnl men; DM had lower rate than stable manual (p < 0.05). Stable non-mnl had lower rate (p < 0.05) than stable mnl. Mobile group rates were between those of stable groups. |

AP = Angina pectoris; BMI = Body mass index; CHD = Coronary heart disease; CVD = Cardiovascular disease; DBP = Diastolic blood pressure; DM = Downwardly mobile; Edu = Education; FEV1 = Forced expiratory volume in 1 second; HOMA = Homeostasis model assessment score; HTN = Hypertension; IHD = Ischemic heart disease; M = Male; MI = Myocardial infarction; Mnl = Manual occupational class; Non-mnl = Non-manual occupational class; Occup = Occupation; RF = Risk factor; RG = Registrar General’s social class categories; RR = Relative risk; SBP = Systolic blood pressure; SES = Socioeconomic status; UM = Upwardly mobile; WHR = Waist-to-hip ratio.

1. Score included the following variables: height, weight, ponderal index, SBP, DBP, pulse, smoking, exercise, CHD history, HTN, more. [↑](#footnote-ref-2)
2. Infant health index composed of sum of # of complications including maternal diabetes, glycosuria, epilepsy, HTN, eclampsia, antepartum hemorrhage, accidental hemorrhage, placenta praevia, previous small baby, higher-risk gestational age, higher-risk birthweight, more. [↑](#footnote-ref-3)
