# Supplementary material for: Evaluating the evidence for models of life course socioeconomic factors and cardiovascular outcomes: a systematic review
Source: BMC Public Health. 2005 Jan 20;5:7. doi: 10.1186/1471-2458-5-7 (PMC548689; doi:10.1186/1471-2458-5-7)
Supplement: Additional File 4 — SES – CVD life course studies using a cumulative SES design [file 1471-2458-5-7-S4.doc]

**Additional file 4. SES—CVD life course studies using a cumulative SES design**

| **1^st^ Author, year & reference #**  **Study name**  **Study size;**  **% male** | **Study design;**  **age at baseline (years)** | **Cumulative SES measure(s)** | **Variables adjusted for other than age** | **CVD risk factor(s) / outcomes measured** | **Key findings** |
| --- | --- | --- | --- | --- | --- |
| Davey-Smith 1997 [82]  Collaborative Study  5645; 100% M | Pros-pective cohort;  35-64 | Sum of # of times at mnl vs. non-mnl SES using father’s, own first, & own current occup class | Smoking, DBP, cholesterol, BMI, FEV1, AP, bronchitis, ischemia | CVD morbidity/ mortality, AP, smoking, height, weight, BP, FEV1, cholesterol | CVD mortality and increasing # of times at mnl SES had positive graded association. Adjusted RR’s for CVD mortality for 1, 2, and 3 life periods of mnl occup vs. none: 1.57 (95% CI: 1.20-2.05), 1.78 (95% CI: 1.37-2.31), 1.92 (95% CI: 1.51-2.45). As # of periods of mnl occup increased, SBP, DBP, BMI, & % smoking increased, while cholesterol, FEV1, & height decreased (for all, p trend < 0.0001). |
| Heslop 2001 [81]  Collaborative Study  958; 0% M | Pros-pective cohort;  35-64 | Sum of # of times at mnl vs. non-mnl SES using father’s, own first, & own current class | DBP, cholesterol. BMI, FEV1, smoking, alcohol, physical activity | CVD mortality, DBP, cholesterol, BMI, FEV1, exercise, smoking, alcohol | Compared to women with 0 or 1 manual occup periods, CVD mortality HR after adjustment for CVD RF’s for women with 2 mnl periods: 1.53 (95% CI: 0.84-2.78), with 3 mnl periods: 1.78 (95% CI: 1.04-3.03), (p trend = 0.036). Significant (p < 0.01) decrease in FEV1 & increase in % smokers as # of manual periods increased. |
| Wamala 2001 [45]  Stockholm Study  584; 0% M | Case-control;  30-65 | Sum of # of instances (0-6) of SES disadvantage: large family, born last, low edu, blue-collar/ housewife, economic hardship | Height, smoking, marriage, physical activity, abdominal obesity, HDL, triglycerides, HTN, fibrinogen | Cases: CHD event (acute MI, unstable/ recurrent AP) | Even after RF adjustment, cumulative lifetime SES disadvantage had strong, graded associations with CHD risk. Adjusted OR for 1, 2, 3-4, and 5-6 instances of disadvantage vs. 0: 1.25 (95% CI: 0.43—3.66), 1.71 (95% CI: 0.61-4.80), 2.63 (95% CI: 0.92-7.56), and 4.08 (95% CI: 1.08-15.35), respectively. Early-life cumulative score: adjusted OR for 3 instances vs. 0: 2.48 (95% CI: 0.90-6.83). Later-life cumulative score, adjusted OR for 3 instances vs. 0: 3.22 (95% CI: 1.02-10.53). |
| Davey-Smith 2002 [52]  Collaborative Study  5628; 100% M | Pros-pective cohort;  35-64 | Sum of # of risks (0-6): Father mnl SES, left edu at < 15 years, current mnl SES, smoking, high alcohol, high deprivation area | None | CVD mortality | CVD mortality RR vs. men with 0 of 6 possible unfavorable risk indicators: 1 unfavorable: 1.98 (1.39-2.82), 2: 2.57 (1.82-3.64), 3: 2.67 (1.89-3.77), 4: 2.83 (2.01-3.98), 5: 4.00 (2.84-5.63), 6: 4.48 (3.06-6.55). A strongly graded risk association was observed. |
| Lawlor 2002 [68]  British Women’s Heart Study  4286; 0% M | Cross-sectional;  60-79 | Cross-classification of father’s longest occup and current occup (RG, mnl/non-mnl) | None | Insulin resistance, HTN, LDL, HDL, BMI, WHR, smoking, triglycerides, alcohol | Being in manual class in both childhood and adulthood, compared to no manual class exposure, associated (p < 0.05) with high insulin resistance, low HDL, high triglycerides, obesity, and smoking. |
| Claussen 2003 [53]  Oslo Mortality Study  101,487; 50% M | Retros-pective cohort;  31-50 | Early life: Index of housing conditions[[1]](#footnote-2) (scored 0-7) Adulthood: standardized income (7 groups by income)[[2]](#footnote-3) | None | CVD mortality | Males at lower SES in childhood and adulthood had higher risk of CVD mortality than those with higher cumulative SES (p trend < 0.05), with indications of a gradient effect. Females did not have cumulatively higher risk. There was interaction (supra-multiplicative) between childhood & adulthood SES, increasing evidence for a cumulative effect. |
| Pensola 2003 [79]  Finnish census cohort  112, 735; 100% M | Retros-pective cohort;  30-42 | Sum of # of times in mnl vs. Non-mnl class, by father’s occup. & own occup at 30-34 (farmers & entrepreneurs excluded) | None | CVD mortality | Increasing risk of CVD mortality by # of times (0-2) in manual class. Significantly higher mortality risk (p < 0.05) for those with 2 manual SES exposures vs. those with none. |

AP = Angina pectoris; BMI = Body mass index; CHD = Coronary heart disease; CVD = Cardiovascular disease; DBP = Diastolic blood pressure; Edu = Education; FEV1 = Forced expiratory volume in 1 second; HR = Hazard ratio; HTN = Hypertension; M = Male; MI = Myocardial infarction; Mnl = Manual occupational class; Non-mnl = Non-manual occupational class; Occup = Occupation; OR = Odds ratio; RF = Risk factor; RG = Registrar General’s social class categories; RR = Relative risk; SBP = Systolic blood pressure; SES = Socioeconomic status; WHR = Waist-to-hip ratio.

1. Housing Index was from 1 (poor) to 7 (well off), and used the following items: dwelling type, # rooms, ownership, telephone, toilet, bath. [↑](#footnote-ref-2)
2. Relative Index of Inequality (RII) used to compare groups both in early life and adulthood. [↑](#footnote-ref-3)
